# Persistence and distribution of dinotefuran in tree of heaven

**DOI:** 10.3389/finsc.2023.1134064

**Published:** 2023-03-23

**Authors:** Justin Keyzer, Phillip Lewis, Deborah G. McCullough

**Affiliations:** ^1^ Department of Forestry, Michigan State University, East Lansing, MI, United States; ^2^ Forest Pest Methods Laboratory, United States Department of Agriculture, Animal and Plant Health Inspection Service, Buzzards Bay, MA, United States; ^3^ Department of Entomology, Michigan State University, East Lansing, MI, United States

**Keywords:** *Ailanthus altissima*, dinotefuran, spotted lanternfly, *Lycorma delicatula*, basal bark spray, insecticide residues

## Abstract

Spotted lanternfly (SLF) (*Lycorma delicatula* (White)), an invasive planthopper discovered in Pennsylvania, U.S.A. in 2014, feeds for approximately six months by sucking phloem sap from trunks and limbs of tree of heaven, *Ailanthus altissima*, along with several native trees and woody vines. Basal trunk sprays of dinotefuran, a systemic neonicotinoid insecticide, are commonly used to reduce SLF densities and spread. Information on dinotefuran persistence and within-tree distribution can help identify optimal timing of annual basal trunk sprays, facilitating efficient use of available resources. We applied dinotefuran to 20 uninfested *A. altissima* trees in early April then periodically sampled foliage to monitor insecticide residues. Foliar dinotefuran residues averaged (± SE) 7.8 ± 1.1 and 6.3 ± 1.2 in July and August, respectively, then dropped significantly to 2.6 ± 0.5 ppm in September. In a second study, 20 A*. altissima* trees were similarly treated with dinotefuran basal trunk sprays in early June. Trees were felled to collect foliage and phloem from branches and the trunk in either mid-July or September. Foliar residues averaged 12.7 ± 1.3 and 14.6 ± 2.2 ppm in July and September, respectively. For trees felled in July, residues were detected in phloem collected from below the spray line on trunks of seven trees and above the spray line on three trees, averaging 8.6 ± 4.4 and 7.4 ± 2.9 ppm, respectively. In trees felled in September, phloem from below spray lines of seven trees averaged 3.7 ± 1.3 ppm but dinotefuran was not detected in phloem from above the spray line on any trees. Dinotefuran was not detected in phloem sampled from any branches in either July or September. Results suggest dinotefuran basal trunk sprays applied between late May and mid June should persist long enough to effectively control SLF late instars and adults.

## 1 Introduction

Spotted lanternfly (SLF), *Lycorma delicatula* (White) (Hemiptera: Fulgoridae), an invasive planthopper native to China and Taiwan, became established in Korea in 2004 (1) and was subsequently detected in the United States in Pennsylvania in 2014. Since then, established populations of SLF have been identified in localized areas of at least 14 states (2). Predictive models based on climate and host distribution suggest SLF could potentially become established across much of the eastern U.S (3, 4). Although SLF adults typically engage in migratory flights and disperse to nearby areas in late summer or fall (5), long distance spread occurs when people accidentally transport SLF life stages into new areas.

Research in field sites in Pennsylvania has confirmed the univoltine life cycle of SLF. Egg hatch begins in mid April and peaks in May (6, 7). Nymphs feed throughout summer, completing four instars. Adults, which first appear in late July, feed intensively in aggregations during their four month life span (8). Mating can occur from early September through late October and oviposition occurs from mid September to early November (7, 8). Each female lays 1-2 egg masses containing 30 to 50 eggs on tree trunks or branches or on hard, solid items including boulders, bricks, outdoor equipment and vehicles (6–9). Egg masses overwinter until hatching begins the following spring.

Adults and all nymphal stages feed on phloem sap, excreting copious amounts of honeydew, which leads to growth of black sooty mold (*Capnodium* spp. [Dothideales: Capnodiaceae]) on host trees, vegetation and outdoor items below infested trees (6, 8). Black sooty mold reduces photosynthetic area of foliage, potentially affecting plant vigor as well as appearance, and contaminating agricultural crops (6, 8, 10). Wasps and ants are often attracted to the sweet honeydew, causing further annoyance to residents in affected areas. Given the relatively long duration of SLF adult activity and the high densities SLF populations can reach, this insect can be a major nuisance for residents in affected areas. To date, SLF is not known to have caused tree mortality, although feeding has killed individual shoots or small branches of black walnut (*Juglans nigra* L. [Fagales: Juglandaceae]), maples (*Acer* spp. [Sapindales: Sapindaceae]) and other native trees. Intensive feeding combined with black sooty mold can also reduce yield, quality or simply render fruit from infested trees, grapevines (*Vitis riparia* Michx [Vitales: Vitaceae]) and hops (*Humulus* spp. [Rosales: Cannabinaceae]) unmarketable (8, 11).

Although SLF can feed on several trees and woody vines, tree of heaven (ToH) (*Ailanthus altissima* (Mill.) [Sapindales: Simaroubaceae]) is the most preferred host for SLF feeding and reproduction (6, 8). ToH, native to China, was introduced into the U.S. in 1784 and was widely planted in urban areas through the 19^th^ century (12). It has subsequently spread across much of the U.S. Today, ToH is considered to be an undesirable invasive because of prolific seed production by female trees and high germination rates (13), its ability to colonize disturbed sites and outcompete more desirable vegetation, and the unpleasant odor of crushed leaves or twigs (14). Tree of heaven can also reproduce clonally *via* sprouts from lateral roots (13, 15, 16) and may root graft with other ToH, monopolizing available nutrients in a site (16). Because ToH is highly intolerant to shade (12, 13, 17), it is rarely present in closed canopy forests but often grows along forest edges.

Early efforts to eradicate or contain SLF in Pennsylvania involved treating male ToH with dinotefuran, a systemic neonicotinoid insecticide commonly applied as a basal trunk spray (18). Although dinotefuran can be applied *via* trunk injection, basal trunk sprays are relatively efficient and can be used on trees that are small or otherwise difficult to treat with trunk injection. High rates of SLF mortality were consistently observed following dinotefuran treatment (19, 20). At the same time, female ToH in areas with SLF infestations were removed or killed with herbicide (18). This encouraged SLF to feed on the treated trap trees and also limited further ToH reproduction.

While SLF eradication is no longer a realistic objective, dinotefuran continues to be widely used for control of SLF in Pennsylvania and more recently infested states (21). Because dinotefuran is highly water-soluble, it is translocated relatively rapidly in trees compared to imidacloprid, another systemic neonicotinoid insecticide, but is less persistent (22–26). For example, in ash (*Fraxinus* spp. [Lamiales: Oleaceae]) trees treated in May, foliar imidacloprid residues continued to increase through the growing season while dinotefuran levels were dropping by late summer (25–27). Recent studies have shown other insecticides, including cover sprays of broad spectrum pyrethroid products, can effectively control SLF nymphs or adults (28, 29). However, given concerns about insecticide drift, impacts on nontarget insects and the difficulty of effectively spraying tall trees, dinotefuran remains an essential tool for SLF management.

Identifying the optimal timing for basal trunk sprays of dinotefuran is an essential aspect of SLF containment and management programs, given that feeding extends for at least six months, and label restrictions prohibit multiple applications in a single year. In a previous study, dinotefuran residues in foliage sampled from ToH treated in May persisted into September, whereas in trees treated in April, residues sharply declined between August and September (20). Spring applications of dinotefuran reduce early instar densities, protecting trees and vines from feeding, honeydew and sooty mold growth. Whether insecticide residues remain adequate to control fourth instars and adults in late summer or autumn when feeding and honeydew production are most intense, however, remains a key question for pest managers. Additionally, when SLF nymphs are not controlled, mature adult females commonly engage in short-range dispersal flights (5, 30), sometimes invading vineyards and orchards where late season insecticide sprays just before or during harvest, are especially problematic. Because resources are rarely sufficient for multiple insecticide applications in a single year, understanding translocation and persistence of dinotefuran can help pest managers efficiently control SLF densities while limiting unnecessary applications.

Systemic insecticides such as dinotefuran are transported in xylem tissue (27, 31) and accumulate in leaves, which function as a major sink for water and nutrients during much of the growing season. Insecticide residues in foliage samples are frequently used to quantify insecticide concentrations, monitor insecticide persistence over time or to compare treatment timing, application methods or other factors. All SLF nymphal stages and adults, however, feed on phloem in tree branches and trunks (6, 8, 32). Observations of high and often rapid SLF mortality following dinotefuran application (19, 20, 33, 34) suggest that either dinotefuran moves into the phloem, i.e., *via* transverse rays, or the mouthparts of SLF insects penetrate phloem and encounter insecticide in xylem vessels. Evaluating dinotefuran presence and concentrations in phloem could help to fully understand options for optimizing SLF control.

We conducted two studies in 2019 to assess dinotefuran persistence and within-tree distribution following basal trunk sprays applied to healthy ToH in sites in Michigan, well beyond any known SLF infestation. In the first study, dinotefuran was applied in early April and residues were quantified in samples of ToH foliage collected periodically until late September when leaves were dropping. Based on previous research and experience, we expected dinotefuran residues would remain relatively high for at least two months before declining in mid to late summer. We also evaluated whether tree diameter affected foliar dinotefuran concentrations at each sampling period. We expected to find little or no relationship between residue levels and tree diameter, given that label application rates are based on tree DBH (diameter at breast height) and the thin outer bark of ToH seemed unlikely to prevent rapid movement of dinotefuran into xylem tissue.

In the second study, we quantified residues in ToH foliage and phloem collected on two post-treatment dates following basal trunk sprays of dinotefuran applied in June. Foliage and phloem samples were collected from trees felled in either July or September to compare dinotefuran levels in the two tissues and to assess potential effects of aspect, sampling dates and tree DBH on dinotefuran concentration. Phloem samples were collected from below and above the spray line on trunks of trees felled in July and trees felled in September. We expected phloem residues below the spray line to decrease over time as insecticide was transported to the canopy but whether dinotefuran would be detectable above the spray line, especially in September, was unknown. Given the many reports of rapid mortality of SLF nymphs and adults on trees treated with dinotefuran (18–21), we anticipated that dinotefuran would be present in phloem from branches, although perhaps at lower levels than in foliage. We also were interested in determining whether the relative sun exposure of leaves and branches affected dinotefuran levels or persistence.

## 2 Materials and methods

### 2.1 Study sites

Study 1 was conducted with ToH growing in an unmanaged, ~0.1 ha strip of land in Lansing, Ingham County, Michigan. The site was in an industrial area with an overstory composed entirely of ToH, and an herbaceous layer of poison ivy (*Toxicodendron radicans* (L.) Kuntze [Sapindales: Anacardiaceae]), and Virginia creeper (*Parthenocissus quinquefolia* (L.) Planch [Vitales: Vitaceae]). On 2 April 2019, 24 ToH trees with DBH ranging from 10.9 to 34.8 cm DBH and averaging 19.3 ± 1.4 cm were selected and tagged. Twenty trees were assigned to a basal trunk spray of dinotefuran and four were left as untreated controls. Brush was cleared around each tree to facilitate access.

Study 2 was conducted in an ~0.4 ha, even-aged plantation of ToH established in 1976 at MSU’s W.K Kellogg Forest in Augusta, Kalamazoo County, MI. A few northern red oak, *Quercus rubra* (L.) [Fagales: Fagaceae] trees grew along the plantation borders while black cherry (*Prunus serotina* (Ehrh.) [Rosales: Rosaceae]) saplings and European buckthorn, (*Rhamnus cathartica* (L.) [Rosales: Rhamnaceae]) grew between and within the rows of ToH. Herbaceous vegetation was dominated by poison ivy, multiflora rose (*Rosa multiflora* (Thunb.) [Rosales: Rosaceae]) and wild raspberry (*Rubus* sp. [Rosales: Rosaceae]) shrubs. On 16 May 2019, brush was cleared at the site (using hand tools) to facilitate access to the trees and to allow a skidsteer to maneuver between and within rows. We tagged and measured DBH of 26 trees across the plantation. Tree DBH ranged from 7.1 to 37.6 cm DBH and averaged 18.8 ± 1.2 cm. Six trees were randomly selected to be left as untreated controls while the remaining 20 were treated with a basal trunk spray of dinotefuran. Even-numbered treated and control trees were felled in mid-summer while odd-numbered treated and control trees were felled in late summer (see below).

Cumulative growing degree days corresponding to each treatment and sampling date were acquired from data recorded by MSU EnviroWeather stations located at the MSU Horticulture Teaching and Research Center, approximately 13 km from the Study 1 site, and from the MSU Kellogg Biological Station, approximately 8 km from the Study 2 site. Cumulative growing degree days were calculated using the Baskerville-Emin method with a base 10°C developmental threshold and a starting date of 1 January. Growing degree day accumulations corresponding to treatment and sampling dates are reported here for potential application in other regions with different weather regimes.

### 2.2 Dinotefuran application

Trees in Study 1 and Study 2 were treated with dinotefuran on 9 April (25 GDD [growing degree days]) and 6 June (291 GDD) 2019, respectively, using the same insecticide rate and application method. Twelve water soluble packets of Transtect^®^ were added to 3.8 liters (one gallon) of distilled water in the tank of a low-pressure 7.5 liter garden sprayer. Formulated insecticide was applied as a basal trunk spray at a rate of 59 ml (2 oz) per 2.54 cm DBH (1.4 g active ingredient per 2.5 cm DBH) to tree trunks from approximately 1.5 m high down to the base, ensuring the entire trunk was covered and the appropriate amount of insecticide was applied. Spray was applied at low pressure to minimize any drift around tree trunks and care was taken to avoid any spray contact with designated control trees.

### 2.3 Sampling

To account for the often irregular crown shape of ToH (17), composite foliage samples from Study 1 trees were comprised of shoots from branches on at least three different aspects, whenever available. Leaf-bearing shoots were clipped from treated and control trees on 25 July (770 GDD),107 days post-treatment. Foliage samples from each tree were placed into labeled bags, returned to the MSU Forest Entomology Laboratory in coolers with blue ice, then frozen. In the lab, leaflets were stripped from petioles, and petioles and woody twigs were discarded. Sampling was repeated on 20 Aug (1077 GDD) and 30 Sept (1425 GDD), at 133 days and 174 days post-treatment, respectively.

For Study 2, half of the trees in the plantation were destructively sampled on 16 July 2019 (771 GDD), 40 days post-treatment. The spray line on each tree trunk was marked, then a skidsteer felled ten of the treated trees and three untreated control trees. Trees were cut at approximately the top of the spray line.

Leaves were collected with hand pruners from canopy branches on three to four aspects of the felled trees, depending on crown structure, and bagged separately by aspect for each tree. Phloem samples were collected from the same canopy branches using drawknives to remove long strips of bark and phloem beginning near the trunk and extending distally until the branch was ≤ 4-5 cm in diameter. Heavy overcast conditions, however, limited our ability to confidently assess relative amounts of sun or shade exposure of individual branches. Drawknives were also used to remove 0.5 to 1.0 m long strips of bark and phloem from the upper half of the trunk on the felled trees. Samples from above the spray line were collected 2.5 to 4 m above ground and samples from below the spray line were collected 0.5 to 1.0 m above ground, within the area that had been sprayed. Phloem readily separated from xylem and outer bark in the branch and trunk samples. Phloem samples from different branches and from above and below the spray line on tree trunks were placed into individual bags. All drawknives and hand pruners used for sampling were sterilized with 70% ethanol between each sample to avoid contamination.

Remaining trees in Study 2 in the plantation were felled and sampled on 17 September (1478 GDD), 103 days post-treatment, using the same methods as above. Exposure to sun, which could presumably affect insecticide concentration or persistence, was qualitatively ranked for each branch that was sampled as 1 if it was fully shaded, 2 if it was partially shaded and 3 if it was fully exposed to sunlight.

Foliage and phloem samples collected in July or in September from the Study 2 trees were bagged, transported in a cooler with blue ice to the MSU Forest Entomology Laboratory, then frozen as in Study 1. Leaflets were stripped from shoots and petioles in the laboratory, then re-frozen. Frozen foliage and phloem samples were shipped overnight to collaborators at the USDA APHIS laboratory in Buzzards Bay, MA on 22 October 2019 for insecticide residue analysis.

### 2.4 Residue analysis

Foliage and phloem samples were removed from bags, air-dried for at least two weeks, then ground in a commercial blender to a fine powder. Blenders were tripled rinsed, scrubbed with soapy water (LIQUINOX^®^ detergent, Alconox Inc., White Plains, NY) and a bottle brush, sprayed with 95% ethanol then rinsed in dionized water to ensure any insecticide residue was removed. Personnel changed nitrile gloves between samples to further minimize any risk of cross contamination.

Analysis of insecticide residues in ToH leaves collected from trees in Study 1 and Study 2, and in ToH phloem from Study 2 trees was determined using commercially available Enzyme Linked Immunosorbent Assay (ELISA) kits (FujiFilm/Horiba; Kyoto, Japan and Wako Chemical, USA Corporation, Richmond, VA). A 0.5 g sample of processed plant material was weighed into a 50 mL plastic centrifuge tube and extracted in 10 mL of pure methanol for 3 hrs on a table-top shaker. Sample tubes were spun down in a high-speed centrifuge for 10 min and the supernatant diluted a minimum of 20x to avoid matrix effects from the kit due to the methanol. Sample aliquots were added to a 96-well plate, developed and the absorbance value calculated according to the manufacturer’s instructions using provided standards of 1.5 ppb to 30 ppb. The effective lower limit of kit detection following sample preparation and dilution is 0.6 ppm.

### 2.5 Statistical analysis

Normality of dinotefuran residues in foliage from Study 1 trees was assessed with a Shapiro-Wilk test and residual plots (PROC MIXED, PROC UNIVARIATE, SAS 9.4) and a square root transformation was applied to normalize residue data (Pr <W = 0.1456).

A one-way ANOVA with repeated measures (PROC MIXED, SAS 9.4) was used to compare differences in foliar dinotefuran residues among the three sample dates with an *a priori* significance level of α = 0.05. The Kenward-Roger correction was used for calculating denominator degrees of freedom because it is more conservative than the MIXED default and is generally recommended for repeated measures analysis to minimize the risk of an increased Type 1 error rate generated by improperly fitted covariance structure. The Tukey-Kramer multiple comparison test was applied when the ANOVA results were significant to identify significant differences among sampling dates. Additionally, linear relationships between foliar dinotefuran residues and tree diameter were assessed with simple linear regression (PROC REG).

Results of a Shapiro-Wilk test and residual plots (PROC UNIVARIATE, SAS 9.4) showed dinotefuran residues in leaf samples collected from the Study 2 trees in the plantation were not normally distributed and data were not normalized by transformation. A two-way nonparametric ANOVA was therefore performed on ranked foliar insecticide residues to assess differences among leaves collected from branches on different aspects of the canopy and between the two sample dates (PROC RANK, PROC MIXED, SAS 9.4). Dinotefuran was not detected in any of the phloem samples collected from branches on either sampling date.

A composite foliar residue value for each tree in Study 2 was calculated by averaging residues in the leaves from the three to four sampled branches in July and again in September. Composite foliar residue values in trees were normal on both sampling dates. An independent t-test was used to assess differences in foliar residues between samples collected from trees felled in July versus September (PROC TTEST, SAS 9.4). Within each month, differences between foliar and trunk phloem residues and between residues in phloem from above and below the spray line were evaluated with paired t-tests. Simple linear regression (PROC REG, PROC UNIVARIATE, SAS 9.4) was applied to assess relationships between foliar residue levels and tree DBH for trees sampled on each date.

## 3 Results

### 3.1 Study 1

**Figure 1:**
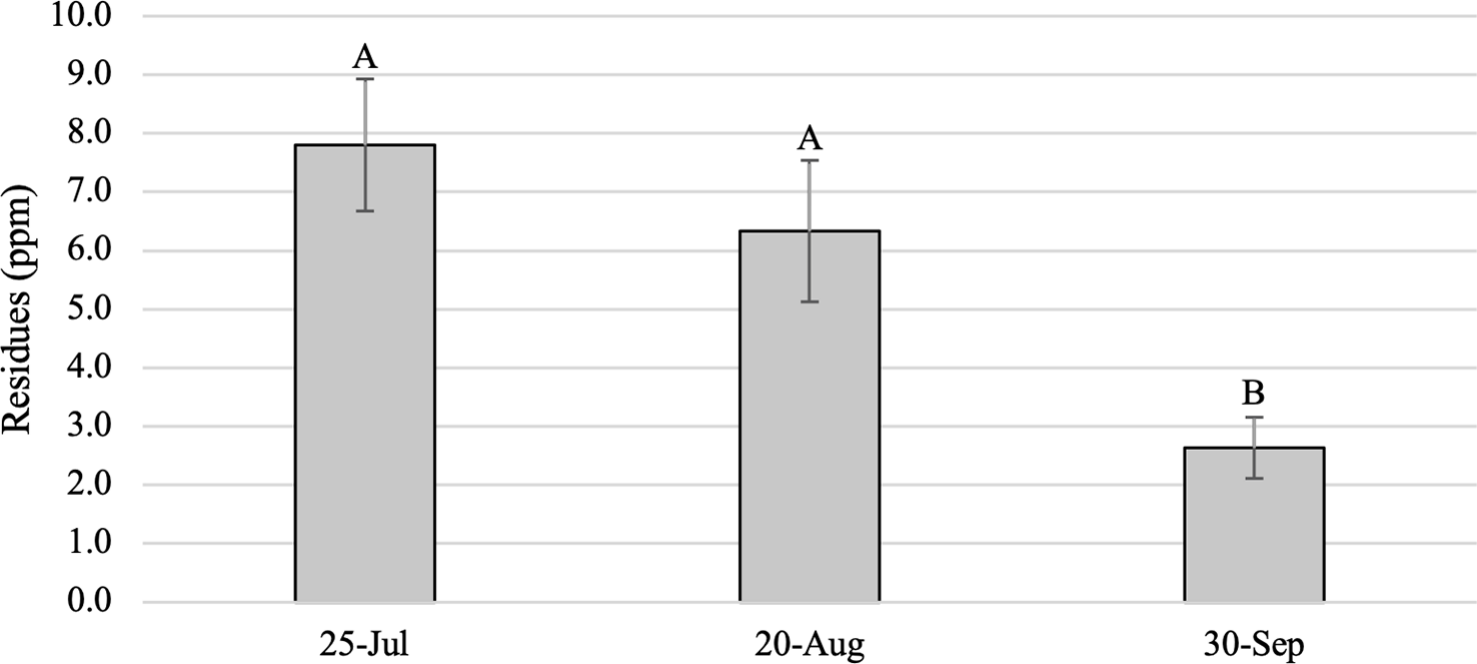
Mean (± SE) dinotefuran residues (ppm) in foliage samples from *Ailanthus altissima* trees in Study 1. Samples were collected periodically in 2019 following a 9 April 2019 basal trunk spray. Letters above bars indicate significant differences (*P* < 0.05) (n = 20 trees).

As expected, foliar dinotefuran residues from Study 1 trees were significantly higher in foliage from treated trees than in untreated controls, which had no dinotefuran, across all months (F = 20.52; df = 1,21.9; *P* < 0.001) and differed among post-treatment sample dates (F = 5.63; df = 2,34.6; *P* = 0.0076) (**Figure 1**). Residues averaged 7.8 ± 1.1 ppm and ranged from 0.7 to 17.0 ppm in July, 6.3 ± 1.2 ppm and 0 to 20.0 ppm in August, and 2.6 ± 0.5 ppm and 0 to 8.8 ppm in September. Residues were significantly higher in samples collected in July (770 GDD) than in September (1425 GDD) (*P* < 0.001) and in August (1077 GDD) compared to September (*P* = 0.0013), but the drop in average dinotefuran concentration between July and August was not significant (*P* = 0.5412) (**Figure 1**). Residues in 14 trees were lower in August than in July, while values increased in the six trees between July and August. Between August and September, residues in 18 of the 20 treated trees had declined and overall residues in September were 50% lower than in August and 66% lower than in July. Residues in two trees increased slightly in September from August, but residue values were substantially lower in these trees from the July values. Tree size did not affect foliar residues in any of the sampling periods; simple linear regressions yielded R^2^ values of 0.02 (*P* = 0.48), 0.03 (*P* = 0.41) and 0.002 (*P* = 0.85) in July, August and September, respectively.

### 3.2 Study 2

Dinotefuran residues were detected in foliage from all treated trees sampled in mid-July (771 GDD), 40 days post-treatment, and in mid-September (1478 GDD), 103 days post-treatment. Mean foliar residue levels averaged 12.7 ± 1.32 and 14.6 ± 2.18 ppm in the ten trees felled and sampled in July and the other ten trees sampled in September, respectively. While average foliar residues were approximately 5% higher in September than in July, the difference was not significant (t = -0.55; df = 1,23; *P* = 0.5895). Results from the two-way ANOVA confirmed the similarity in foliar residues between samples collected in July and September (F = 0.40; df = 1,87; *P* = 0.53). Residues also did not differ among foliage samples collected from branches at different aspects (F = 0.02; df = 3,87; *P* = 0.89). Mean foliar residues ranged from 11.9 ± 1.90 ppm (eastern aspect) to 13.5 ± 2.69 ppm (southern aspect) in July and from 12.4 ± 3.78 ppm (southern aspect) to 16.3 ± 5.50 ppm (northern aspect) in September. As in Study 1, tree DBH did not affect mean foliar residues in trees sampled in either July (R^2^ = 0.16; *P* = 0.25) or September (R^2^ = 0.0002; *P* = 0.97). Leaves from one tree sampled in September exhibited an unusually high dinotefuran concentration but excluding this outlier had little effect on results (R^2^ = 0.03; *P* = 0.621). All branches that were sampled to collect foliage and phloem from trees felled in September were either partially (Rank 2) or fully exposed to sun (Rank 3). There was no evidence that sun exposure affected foliar dinotefuran residues.

Dinotefuran residues in phloem samples collected from above and below the spray line on trunks of the felled trees varied substantially and were often too low to be detected. Phloem samples collected in July from below the spray line yielded detectable dinotefuran residues in seven of the ten felled trees, averaging 8.6 ± 4.4 and ranging from 0.8 to 32.2 ppm. Three trees had detectable levels of dinotefuran in phloem from above the spray line, with concentrations ranging from 1.6 to 10.6 ppm and averaging 7.4 ± 2.9 ppm. Phloem from only one tree had detectable dinotefuran residues in samples from both above (9.8 ppm) and below the spray line (0.8 ppm).

In September, none of the phloem samples collected from above the spray line on the trunks of the ten felled trees had detectable dinotefuran residues. Seven of these trees had measurable dinotefuran residues in phloem from below the spray line, ranging from 0.8 to 10.8 and averaging 3.7 ± 1.3 ppm.

Overall, residues in phloem samples collected from tree trunks were significantly lower than residues in foliage collected from the same trees in July (t = 3.60; df = 9; *P* = 0.0058) and September (t = 4.31; df = 9; *P* = 0.002). On average, phloem residues in samples from below the spray line were 53% lower than foliar residues in July and 83% lower than September foliar residues. Phloem residues in samples from above and below the spray line did not significantly differ in July (t = 0.99; df = 9; *P* = 0.35) but were significantly higher below the spray line than above the spray line in September (t = 2.49; df = 9; *P* = 0.03). None of the phloem samples collected from branches had detectable dinotefuran residues, regardless of sample date or aspect.

## 4 Discussion

Basal trunk sprays of dinotefuran remain a key tool for managing SLF infestations to reduce insect density, protect the health of trees and other hosts, and lessen the annoyance or anxiety experienced by residents during outbreaks. Identifying the optimal time to apply dinotefuran, however, remains an essential question for pest managers dealing with established SLF populations along with newly discovered infestations. Regulatory personnel, IPM specialists and resource managers desire a high level of SLF control that is also cost-effective and logistically practical (21). Launching dinotefuran applications in spring could be advantageous when extensive areas require treatment, especially if personnel or funding are likely to be limited later in the season. Reducing densities of SLF early instars in an area also decreases feeding and honeydew production by later life stages, minimizing potential injury to host plants. Conversely, in other situations, SLF infestations may not be discovered until late summer or autumn when brightly colored 4^th^ instars or the large adults are more easily observed. High densities of SLF can also appear in previously uninfested areas following migratory flights by mature adults in late summer or fall (5).

In our studies, as in most research with systemic insecticides, residues in samples of foliage were quantified to evaluate persistence of dinotefuran. Sampling leaves to assess insecticide concentrations causes minimal injury to trees and facilitates repeated sampling over time. Tree DBH, which ranged from 10.9 and 7.1 cm up to 34.8 and 37.6 in Study 1 and Study 2, respectively, did not affect foliar dinotefuran residues in any sampling period. This is not surprising since the amount of insecticide applied to any tree is based on the DBH of the tree. Previous studies with ash trees, which are ring porous like ToH, have shown that systemic insecticides are carried in xylem vessels in the outer ring of sapwood from the trunk to the canopy, where expanding buds and leaves act as a strong sink for xylem (27, 31, 35). It is notable, however, that the thicker outer bark on large trees relative to the smaller trees in this study did not limit absorption nor affect translocation of dinotefuran applied *via* basal trunk sprays in early April (Study 1) or June (Study 2). Age of the largest trees in Study 1 are unknown, but records show that the mature trees in the plantation used for Study 2 were 48 years old at the time of treatment and sampling. A high proportion of trees in most areas where SLF is established will likely be of similar size and can be efficiently treated with basal trunk sprays instead of more laborious trunk injections. Since ToH trees can reportedly attain diameters of >1.5 m (14), however, further evaluation of insecticide translocation in very large trees may be warranted.

While foliar residues are ideal for monitoring insecticide presence over time or comparing different treatments, SLF feeds by sucking phloem sap from tree branches, trunks, and woody vines. Several studies have reported high and relatively rapid SLF mortality following dinotefuran application (19, 20, 28, 33, 34), indicating that these insects must encounter lethal levels of insecticide as they feed. We anticipated dinotefuran residues would be relatively high in phloem samples collected from below the spray line on tree trunks, e.g., the area where the dinotefuran spray was physically applied. We also expected to detect some level of dinotefuran in phloem samples from above the spray line and in branches, indicative of dinotefuran translocation to the canopy. Movement of dinotefuran from xylem into phloem *via* transverse rays could presumably result in the consistently high SLF mortality observed on treated trees (19, 20). However, in the ten Study 2 trees sampled in July, only 40 days post-treatment, dinotefuran was undetectable below the spray line in three trees and above the spray line in seven of the trees. The lack of detectable dinotefuran residues in phloem from any of the branches sampled on the Study 2 trees was also unexpected, particularly given the insecticide levels in leaves from those same branches. It is possible that dinotefuran in the phloem samples from the branches was present at concentrations below the detection limit of 0.6 ppm of our assay. While LC_50_ values for dinotefuran corresponding to SLF mortality are unknown, it seems unlikely that residues consistently below detection limits would cause the high SLF mortality rates previously observed in multiple infestations (19, 20).

A possible mechanism to account for these seemingly contradictory observations is that while SLF need to access nutrients in phloem, their mouthparts may penetrate phloem and reach xylem tissue in the outer sapwood ring, which could result in the insects encountering a lethal dose of insecticide. Research has suggested that phloem-feeding emerald ash borer (EAB) (*Agrilus planipennis* Fairmaire [Coleoptera: Buprestidae]) larvae may similarly encounter insecticide when early instar galleries score the outer xylem in ash trees (26, 35). Further research into the mechanics of SLF feeding is needed to understand how these insects encounter systemic insecticides, particularly small early instars with short stylets (36).

When young ash (*Fraxinus* spp.) trees were injected with ^14^C-labelled imidacloprid, another systemic neonicotinoid, residues in subsequent foliage samples varied depending on the position of branches relative to injection sites, and with the height of branch whorls (31). Translocation patterns of ToH and ash, both ring porous trees, are probably similar but in our Study 2 trees, foliar residues were not affected by aspect of the leaf-bearing branches we sampled. Basal trunk sprays, which are applied around the entire circumference of the tree, may facilitate a more even distribution of insecticide throughout the canopy than trunk injection. Additionally, we hypothesized that higher transpiration rates in leaves fully exposed to sun could result in more rapid translocation or higher residues, at least initially, than in shaded branches. However, we found no evidence that exposure to sunlight affected insecticide translocation rates or persistence in foliage. Virtually all foliage-bearing branches on trees in both Study 1 and Study 2 were at least partially exposed to sunlight, while branches below the canopy or those that were shaded by adjacent trees were dead, a pattern consistent with the low shade tolerance exhibited by ToH, and its rarity in closed canopy forests (17, 37).

Although dinotefuran LC_50_ values for SLF have not been determined, we assumed that trees with high dinotefuran concentrations in leaves would be more toxic to SLF nymphs and adults than trees with lower residues. Foliar residues from Study 2 trees, treated in June (291 GDD), averaged 12.8 ± 1.3 and 14.6 ± 2.2 at 40 and 103 days post-treatment, respectively, while residues in Study 1 trees, treated in early April (25 GDD), averaged 7.8 ± 1.1 and 6.3 ± 1.2 ppm in samples collected in July and August, 107 and 133 days post-treatment, respectively. Generally lower foliar residues in Study 1 trees compared with Study 2 trees may reflect the poor Study 1 site conditions, reflected in lower respiration and translocation rates. Study 1 trees were in a narrow, highly disturbed strip of land bordered by parking lots, while Study 2 trees were on a relatively high quality site with minimal disturbance. Variability in foliar residues among Study 1 trees, as evidenced by standard errors, increased between July and August (albeit slightly), and between July and Sept for Study 2 trees, a pattern consistent with differences among trees in insecticide translocation. Increased foliar residues in Study 2 trees between 40 and 103 days post-treatment presumably reflects continued translocation of insecticide from the lower trunk to canopy branches and leaves. Six of 20 Study 1 trees had higher residues in mid August (133 days post-treatment; 1077 GDD) than in late July (771 GDD), indicating translocation of insecticide was still occurring between 107 and 133 days post-treatment in some trees.

Collectively, these results suggest basal trunk sprays should provide effective control of SLF for at least 100 days and probably for as much as 135 days post-treatment in most trees. However, residues dropped sharply in Study 1 trees during the 41 days between the mid August and late September samples, when residues averaged < 3 ppm (174 days post-treatment). Similarly, the number of Study 1 trees with relatively low foliar residues, e.g., ≤ 5 ppm, increased from seven trees in the July samples, to 12 trees in August and 18 trees in September. In an earlier study, dinotefuran residues in trees treated in mid to late May remained relatively stable in September (20).

Early season treatments to reduce densities of early instars would presumably limit feeding, honeydew production and associated impacts in a given area throughout the summer. However, applications made too early will likely result in trees with relatively low and rapidly declining residues from late August through October, a period when SLF adult feeding, dispersal and migratory flights are likely to peak (38). Delaying dinotefuran basal trunk sprays until late May or mid June should provide effective control of late instars and SLF adults in October, although early instar feeding and local dispersal would still occur. Understanding more about translocation and persistence of dinotefuran and other systemic insecticides including imidacloprid would be valuable for SLF programs and more broadly for insect pests of other trees.

## Data availability statement

The raw data supporting the conclusions of this article will be made available by the authors, without undue reservation.

## Author contributions

DM and PL conceived the project, acquired funding, and designed the experiments. DM oversaw the experiments, coordinated and participated in field work including insecticide applications, sampling and data collection. PL directed sample analysis of the insecticide residues. JK assisted with field work, processed samples and analyzed data. JK and DM drafted the manuscript with contributions from PL. All authors contributed to the article and approved the submitted version. 
